# Planar Hall Effect Magnetic Sensors with Extended Field Range

**DOI:** 10.3390/s24134384

**Published:** 2024-07-05

**Authors:** Daniel Lahav, Moty Schultz, Shai Amrusi, Asaf Grosz, Lior Klein

**Affiliations:** 1 Department of Physics, Institute of Nanotechnology and Advanced Materials, Bar-Ilan University, Ramat-Gan 52900, Israel; daniellahavca@gmail.com (D.L.);; 2 Department of Electrical and Computer Engineering, Ben-Gurion University of the Negev, P.O. Box 653, Beer-Sheva 84105, Israel

**Keywords:** planar hall effect, magnetic sensor, equivalent magnetic noise, magnetometer

## Abstract

The magnetic field range in which a magnetic sensor operates is an important consideration for many applications. Elliptical planar Hall effect (EPHE) sensors exhibit outstanding equivalent magnetic noise (EMN) on the order of $\mathrm{pT}/\sqrt{\mathrm{Hz}}$, which makes them promising for many applications. Unfortunately, the current field range in which EPHE sensors with $\mathrm{pT}/\sqrt{\mathrm{Hz}}$ EMN can operate is sub-mT, which limits their potential use. Here, we fabricate EPHE sensors with an increased field range and measure their EMN. The larger field range is obtained by increasing the uniaxial shape-induced anisotropy parallel to the long axis of the ellipse. We present measurements of EPHE sensors with magnetic anisotropy which ranges between 12 Oe and 120 Oe and show that their EMN at 10 Hz changes from 800 $\mathrm{pT}/\sqrt{\mathrm{Hz}}$ to 56 $\mathrm{nT}/\sqrt{\mathrm{Hz}}$. Furthermore, we show that the EPHE sensors behave effectively as single magnetic domains with negligible hysteresis. We discuss the potential use of EPHE sensors with extended field range and compare them with sensors that are widely used in such applications.

## 1. Introduction

Magnetic field sensing plays a pivotal role in numerous technological applications, including automotive [1,2,3,4], biomedical [5,6,7], industrial automation [8], and diagnostics applications [9], among others [10,11,12,13].

Among all magnetic sensors, planar Hall effect (PHE) sensors offer advantages such as equivalent magnetic noise (EMN) on the order of $\mathrm{pT}/\sqrt{\mathrm{Hz}}$, design simplicity, low cost, and compatibility with integrated circuit fabrication processes [14,15,16]. A recent comprehensive review of PHE sensors [17] highlights the increasing interest in PHE sensors and shows that there are still numerous paths for further advancements that will make them even more attractive for different applications, including in microfluidics [18,19] and flexible sensorics [20].

In addition to the many advantages of PHE sensors, there is also a disadvantage, as typically the field range of sensitive PHE sensors is on the order of hundreds of μT [20,21,22], which is a limiting factor for some important applications (e.g., in the automotive industry). Therefore, to address the growing need for sensitive magnetic sensors with a large measuring field range in various industrial, scientific, and consumer applications, it is important to extend the field range of PHE sensors and study the effect of such an extension on their EMN.

Different methods have previously been used to increase the field range of magnetoresistive sensors, including modifications of the ferromagnetic layer [23] and variations in the thicknesses of spacer and capping layers [24,25]. Here, on the other hand, we achieve this goal by manipulating their shape-induced magnetic anisotropy.

In this study, we use elliptical PHE (EPHE) sensors which exhibit uniaxial anisotropy dominated by their shape anisotropy [26]. For elongated ellipses, their shape-induced anisotropy is proportional to the ratio of their film thickness to the short axis of their ellipse, thus we fabricate elliptical sensors of various sizes while keeping constant the film thickness and ratio between the principal axes of the ellipse. We fabricated EPHE sensors with the size of their short axis ranging from 200 microns to 20 microns and achieved a tenfold increase in the magnetic anisotropy field, from approximately 12 Oe to 120 Oe. This modification broadens their operational field range from 4 Oe to 40 Oe, while the EMN at 10 Hz degrades from 800 $\mathrm{pT}/\sqrt{\mathrm{Hz}}$ to 56 $\mathrm{nT}/\sqrt{\mathrm{Hz}}$. The change in magnetic anisotropy fits the theoretical model for shape-induced anistropy well, and the obtained EMN indicates that the noise above 10 Hz of EPHE sensors of different sizes is quite similar when they are operated using the same current density. We also demonstrate that these EPHE sensors behave effectively as single magnetic domains with negligible hysteresis. We address possible routes for further improvement of the EMN while maintaining this larger operational field range and compare the performance of EPHE sensors with extended field ranges with the features of sensors commonly used in applications where sensors are expected to operate in fields in the mT range.

## 2. Principle and Design

### 2.1. Magnetoresistance

Ferromagnetic materials exhibit a phenomenon called anisotropic magnetoresistance (AMR), in which their resistance changes depending on the orientation of their magnetization (**M**) relative to the direction of an applied electric current density (**J**). This effect is caused by a spin–orbit interaction, which influences the scattering of electrons in the material. The exact mechanism behind this effect can vary depending on the specific characteristics of the material.

This phenomenon gives rise to a transverse voltage as a function of the angle θ between **M** and **J**. This effect is called PHE as the magnetization, the electric current, and the transverse electric field are in the same plane.

The AMR and PHE manifest as longitudinal ($\rho_{xx}$) and transverse ($\rho_{xy}$) resistivities, respectively. In amorphous magnetic films, the dependence of $\rho_{xx}$ and $\rho_{xy}$ on the angle θ between the current and the magnetization is given by

(1) $$\rho_{xx}=\rho_{⊥}+\left(\rho_{\|}-\rho_{⊥}\right)\cos^{2}\theta$$

(2) $$\rho_{xy}=\left(\rho_{\|}-\rho_{⊥}\right)\sin\theta \cos\theta$$

where $\rho_{\|}$ and $\rho_{⊥}$ are the resistivities of the current parallel and perpendicular to the magnetization, respectively.

In order to use the PHE for sensing, the magnetization of the magnetic conductor should be uniform and change its orientation predictably, reversibly, and without hysteresis in response to an applied magnetic field. To achieve this behavior, the conductor should have magnetic anisotropy, typically with an easy axis that is parallel to the direction of the current.

### 2.2. The Stoner–Wohlfarth Model

**Figure 1:**
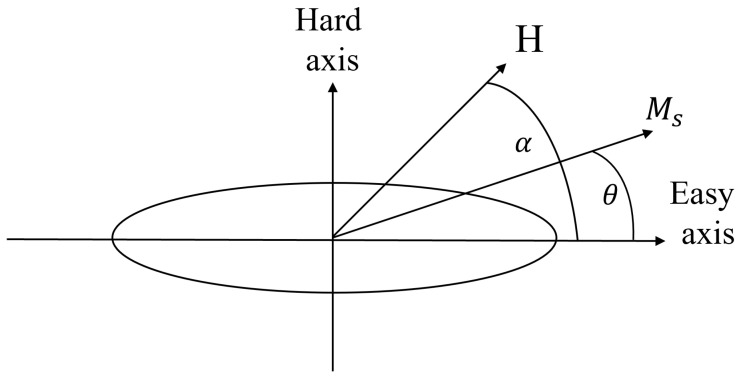
An illustration of a single magnetic domain with an ellipse shape under an external magnetic field **H** applied at an angle α with respect to the easy axis. The magnetization in this case is rotated by an angle of θ from the easy axis.

This model describes magnetization behavior in the simplest case of a ferromagnetic particle with uniaxial magnetic anisotropy that is uniformly magnetized in the presence of an external magnetic field. Applying an external magnetic field **H** at an angle α with respect to the easy axis (EA) will tilt the magnetization to an angle θ with respect to the EA (Figure 1).

The angle θ is determined by minimization of the Stoner–Wohlfarth (SW) Hamiltonian, which is

(3) $$H=\underset{I}{\underset{︸}{K_{u}\sin^{2}\theta }}-\underset{\mathrm{II}}{\underset{︸}{HM_{s}\cos\left(\alpha -\theta \right)}}$$

where $K_{u}$ is the magnetic anisotropy constant, **I** is the contribution of the uniaxial anisotropy (shape), and **II** is due to the interactions between the external field **H** and the saturated magnetization **M_s_**.

### 2.3. Planar Hall Effect Sensors

#### 2.3.1. Elliptical Planar Hall Effect Sensors

**Figure 2:**
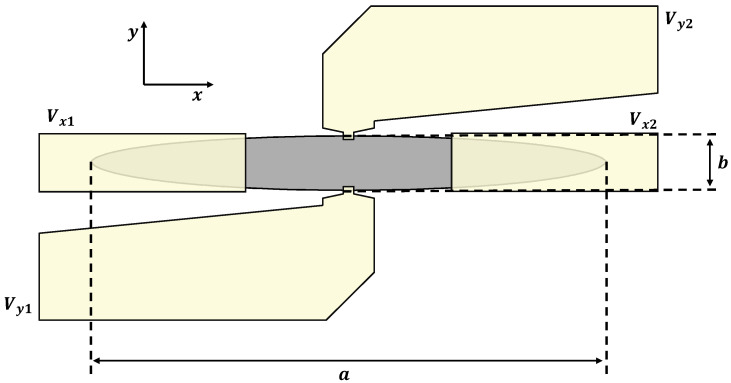
Schematics of the sensor’s geometry (not to scale). The long and short axes of the ellipse are labeled as *a* and *b*, respectively. The yellow regions are the gold electrical contact pads. The sensor is excited between $V_{x1}$ and $V_{x2}$, and the signal is measured across $V_{y1}$ and $V_{y2}$.

The shape of EPHE sensors, as illustrated in Figure 2, induces uniaxial magnetic anisotropy aligned with the long axis (i.e., the easy axis). When these sensors are exposed to an external magnetic field, their response is well described by the Stoner–Wohlfarth Hamiltonian. In the case of an ellipse with thickness *t* and principal axes *a* and *b* (where a≫b≫t), the shape anisotropy is given by [26]

(4) $$H_{s}∼4\pi M_{s}\frac{t}{b}∼10,807\frac{t}{b}\left[Oe\right]$$

where $M_{S}∼800\left[\frac{\mathrm{emu}}{{\mathrm{cm}}^{3}}\right]$ for Permalloy (Py).

Namely, the anisotropy field of elongated EPHE sensors is determined by the thickness (*t*) [27] and the size of the hard axis (*b*) [16]. Therefore, either reducing the length of the hard axis or increasing the thickness will increase the magnitude of the shape induced anisotropy of the sensor and, hence, the magnetic field range in which it can operate.

We note that our EPHE sensors have two primary sources of magnetic anisotropy: field-induced anisotropy ($H_{g}$), which is associated with the magnetic field present during the growth process (see Section 3.1 below), and shape anisotropy ($H_{s}$), as mentioned above. Consequently, the effective anisotropy field ($H_{eff}$) is the sum of both growth and shape anisotropies.

#### 2.3.2. Sensitivity

The sensitivity of an EPHE sensor is defined as the ratio between its PHE voltage $V_{y}$ and its applied magnetic field in the *y* direction, $H_{y}$, for a given current $I_{x}$ flowing parallel to the easy axis. When $H_{y}$ is small compared to the effective anisotropy field $H_{eff}$, the sensitivity is given by [26,28]

(5) $$S_{y}=\frac{V_{y}}{H_{y}}=I_{x}\frac{▵\rho }{t}\frac{1}{H_{eff}}$$

where ▵ρ is the sensor resistivity anisotropy ($▵\rho =\rho_{\|}-\rho_{⊥}$).

To determine the sensitivity ($S_{y}$), the transverse voltage is measured between $V_{y1}$ and $V_{y2}$ while applying an in-plane magnetic field along the y-axis, with a current of $I_{x}$ flowing between $V_{x1}$ and $V_{x2}$ (Figure 2).

According to Equation (5), the sensitivity, $S_{y}$, is proportional to the current, $I_{x}$, and inversely proportional to the effective anisotropy field, $H_{eff}$. Consequently, decreasing the ellipse size by a factor of *n* while maintaining the same current density will increase the shape anisotropy by *n* while decreasing $I_{x}$ by the same factor. Therefore, we expect the sensitivity of the sensor to decrease by $n^{2}$.

#### 2.3.3. Equivalent Magnetic Noise

The equivalent magnetic noise (EMN, sometimes referred to as resolution) of the sensor [15,28,29] is defined as

(6) $$EMN\left(f\right)=\frac{e_{\sum }\left(f\right)}{S_{y}}$$

where $S_{y}$ and $e_{\sum }$ are the sensitivity (given by Equation (5)) and the total noise spectral density (TNSD), respectively.

The TNSD, $e_{\sum }$, has three main components: 1/f noise, thermal noise (both originating from the sensor), and preamplifier noise. Namely,

(7) $$e_{\sum }\left(f\right)=\sqrt{e_{1/f}^{2}+e_{thermal}^{2}+e_{amp}^{2}}$$

The 1/f noise is dominant at low frequencies, while the thermal noise, often referred to as white noise, is dominant at high frequencies.

The 1/f noise of the sensor is described by

(8) $$e_{1\;/\;f}=\sqrt{V_{x}^{2}\frac{\delta_{H}}{N_{c}\cdot Vol\cdot f^{\beta }}}$$

where $V_{x}$ denotes the bias voltage, $\delta_{H}$ is the Hooge’s parameter, $N_{c}$ represents the free electron density of the ferromagnetic layer (e.g., Permalloy), *f* is the frequency, β is a constant, and Vol is the effective volume contributing to electron conduction in the material.

Thermal noise, also known as Johnson noise, results from the thermal agitation of electrons in the conductor and can be expressed as

(9) $$e_{thermal}=\sqrt{4k_{B}TR_{y}}$$

where $k_{B}$ is the Boltzmann constant, *T* is the absolute temperature, and $R_{y}$ is the resistance of the sensor measured across the y terminals.

The preamplifier noise, which includes contributions from voltage noise and current noise, referred to as the amplifier’s input, is calculated by

(10) $$e_{amp}=\sqrt{v_{amp}^{2}+{\left(R_{y}i_{amp}\right)}^{2}}$$

where $v_{amp}$ and $i_{amp}$ are the voltage noise and current noise of the operational amplifier, respectively.

### 2.4. Design and Materials

We fabricate EPHE sensors with a thickness of 200 nm and hard axis lengths of 200, 100, 50, and 20 microns.

We use Permalloy (${\mathrm{Ni}}_{80}{\mathrm{Fe}}_{20}$, Py) as the ferromagnetic (FM) layer due to its large PHE and low coercive field. Tantalum (Ta) serves a dual purpose as a seeding layer (SL) and a capping layer (CL). Ta’s remarkable ability to align the crystal structure of a growing film with its own promotes the formation of a more uniform and ordered film, thereby improving crystallographic ordering and reducing magnetic coercivity. Additionally, a capping layer of Ta protects the Py film from oxidation.

## 3. Fabrication and Characterization

### 3.1. Fabrication

**Figure 3:**
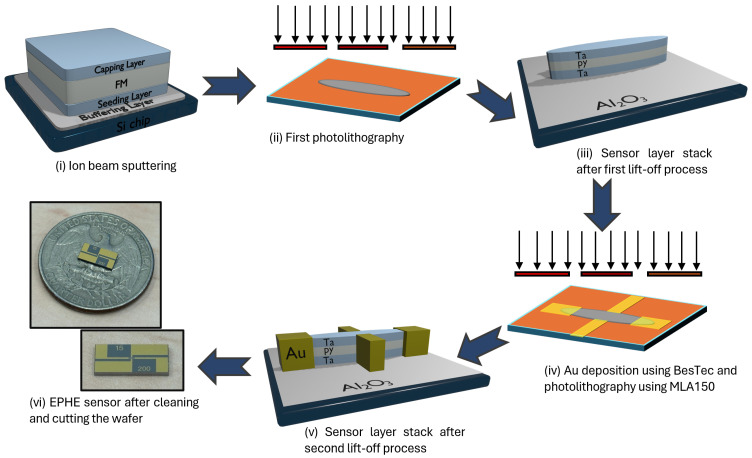
An illustration of the fabrication process of the EPHE sensor.

Figure 3 illustrates the fabrication process. Initially, ${\mathrm{Al}}_{2}O_{3}$(35 nm)/Ta(15 nm)/Py(200 nm)/Ta(15 nm) are deposited on a wafer using an Ion Beam Sputtering (IBS) system [30]. To induce uniaxial anisotropy, permanent magnets are arranged in a Hallbach array configuration on the holder to produce a uniform in-plane magnetic field of 100 Oe during deposition [15,30,31]. Next, elliptical shapes with major axis (*a*) and minor axis (*b*), with an aspect ratio 1:8, are patterned using photo-lithography, ion milling, and wet etch processes. Gold contacts are then added using a second round of photo-lithography and lift-off processes. Finally, the wafer is cleaned and diced into individual sensors using a DISCO dicing machine.

### 3.2. Measurement Set-Ups

#### 3.2.1. Magnetic Characterization

**Figure 4:**
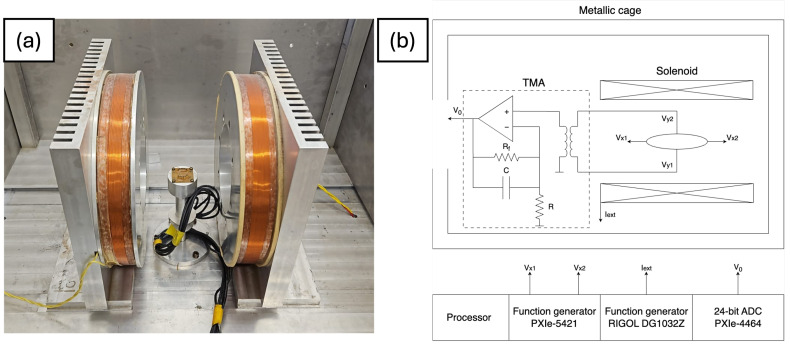
(**a**) Magnetic characterization system. (**b**) Schematic of the noise measurement system.

Our magnetic characterization system (shown in Figure 4a) consists of two coils that generate an x–y plane magnetic field up to 180 Oe. The sample under study is excited with a current source (Keithley 6221), and measured using a nanovoltmeter (Keithley 2182A), while it sits on a stage that rotates using a brushless motor and gear system. The stage has an angular resolution of 0.03° and rotates more than 360°. The entire system is housed in a metallic cage to reduce noise and is controlled by a computer using Matlab. Using this system, we measure $H_{eff}$ and $▵R_{PHE}$, where $▵R_{PHE}=▵\rho \;/\;t$.

#### 3.2.2. Noise Characterization

We use an ac noise measurement set up (shown on Figure 4b) to measure the sensitivity and EMN of the device.

The sensor is excited with an ac current at 1210 Hz along the magnetic easy axis (the long axis of the ellipse) using a NI-PXIe-5421 (National Instruments, Austin, TX, USA) function generator. The PHE voltage is amplified using an ultra-low noise transformer-matched amplifier (TMA) and digitized using NI-PXIe-4464 (National Instruments, Austin, TX, USA), an analog-to-digital converter [29]. The use of an ac source allows us to modulate the output voltage of the sensor and avoid preamplifier 1/f noise. Data acquisition and system control are implemented using National Instruments (NI) DAQ modules and interfaced with a lab computer through a home-made NI-LabVIEW program platform.

Sensitivity is measured by applying a weak, low-frequency magnetic field generated by a solenoid, which is situated within a magnetic shield. For the characterization of noise, the sensor itself is measured inside a 7 layers magnetic shield which suppresses the environmenntal magnetic noise.

## 4. Results and Discussion

**Figure 5:**
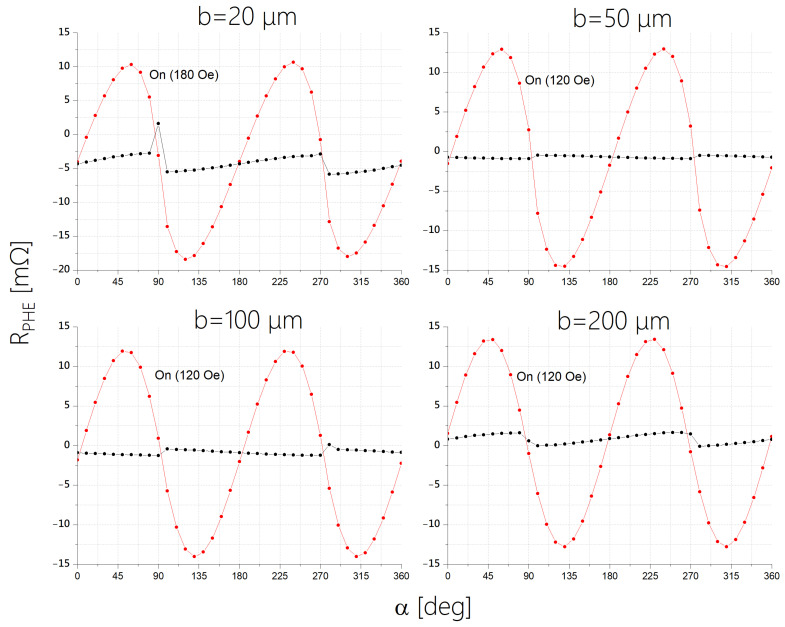
Demonstration of the effective single-domain behavior of EPHE sensors. The PHE is measured as a function of the angle α between *H* and *I*. The current is applied along the long axis of the ellipse. For each α, the voltage is measured twice: with H = 120 Oe (red) and with H = 0 (black). For a sensor with b=20 microns, we apply a magnetic field of 180 Oe by adding ferromagnetic cores, as a field of 120 Oe is insufficient to align the sensor’s magnetization with the applied field direction.

Figure 5 demonstrates effective single-domain behavior. When the magnetization is tilted away from the easy axis by an external field, it fully returns to the easy axis once the field is removed. This behavior is evidenced by measuring the PHE with and without an external field. The small variations in the zero-field values are associated with the change in the relative orientation of a small ambient field relative to the easy axis.

By reducing the dimension of the hard axis, *b*, while keeping the ratio between a and b, we extend the field range of the EPHE sensor, as noted in Table 1.

We note that, for a constant current density, the sensitivity of the 20-micron sensor is approximately 100 times lower than that of the 200-micron sensor. This is in close agreement with the theoretical predictions discussed in Section 2.3.2.

**Figure 6:**
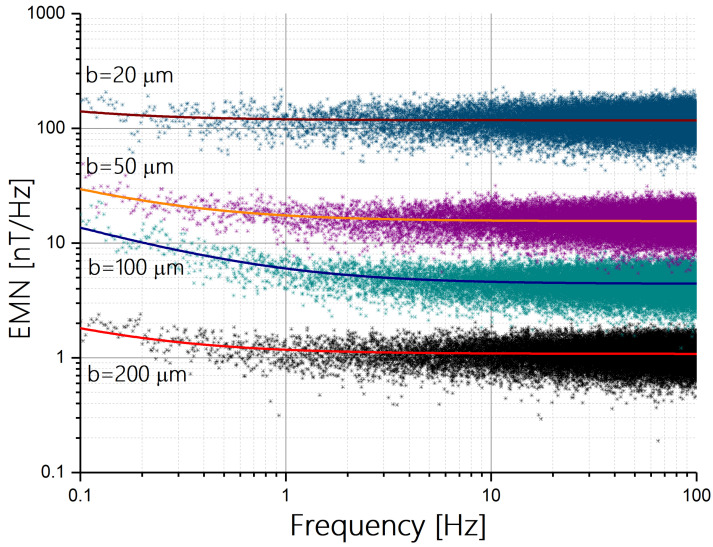
Equivalent magnetic noise (EMN) of the PHE sensors versus frequency for different hard axis values and the same current density.

Figure 6 presents the EMN from 0.1 to 100 Hz for sensors with different hard axis values. The EMN is fitted with an $EMN\left(f\right)=\sqrt{B^{2}+\frac{A^{2}}{f}}$, where A and B are fit parameters [15].

For high frequencies (f≥10 Hz), the noise of the sensors can be approximated by

(11) $$e_{\sum }\left(f\geq 10\;\mathrm{Hz}\right)=\sqrt{e_{thermal}^{2}+e_{amp}^{2}}$$

Thus, the EMN of the sensors at high frequencies is

(12) $$EMN\left(f\geq 10\;\mathrm{Hz}\right)=\frac{e_{\sum }\left(f\geq 10\;\mathrm{Hz}\right)}{S_{y}}=\frac{\sqrt{e_{thermal}^{2}+e_{amp}^{2}}}{I_{x}\frac{▵\rho }{t}\frac{1}{H_{eff}}}$$

As the amplifier noise, $e_{amp}\approx 200\;\mathrm{pV}/\sqrt{\mathrm{Hz}}$ [29], and the thermal noise are of the same order, we expect (and observe) similar high-frequency noise for our sensors. Consequently, the change in EMN is mostly associated with the change in sensitivity. As discussed in Section 2.3.2, if the in-plane dimensions of the sensor are reduced by a factor *n* and it is operated with the same current density, the sensitivity is expected to decrease by $n^{2}$, leading to an increase in the EMN by a factor of $n^{2}$.

As depicted in Figure 6, at a frequency of 10 Hz, we obtain EMNs of approximately 1.1 $\mathrm{nT}\;/\;\sqrt{\mathrm{Hz}}$, 4.4 $\mathrm{nT}/\sqrt{\mathrm{Hz}}$, 16 $\mathrm{nT}/\sqrt{\mathrm{Hz}}$, and 117 $\mathrm{nT}/\sqrt{\mathrm{Hz}}$ for sensors with hard axis values of 200, 100, 50, and 20 microns, respectively. The data clearly indicate that the EMN of each sensor scales roughly as $n^{2}$. Specifically, the EMN of the 20-micron sensor is approximately 100 times larger than that of the 200-micron sensor, as expected.

Using trial and error, we found that it is possible to improve the EMNs of the sensors with hard axes of 20 and 50 microns by doubling the current density used in the EMN measurements presented in Figure 6. We note that, at a frequency of 10 Hz, the 20-micron and 50 micron sensors attain an EMN of 56 $\mathrm{nT}/\sqrt{\mathrm{Hz}}$ and 7.7 $\mathrm{nT}/\sqrt{\mathrm{Hz}}$, respectively.

**Figure 7:**
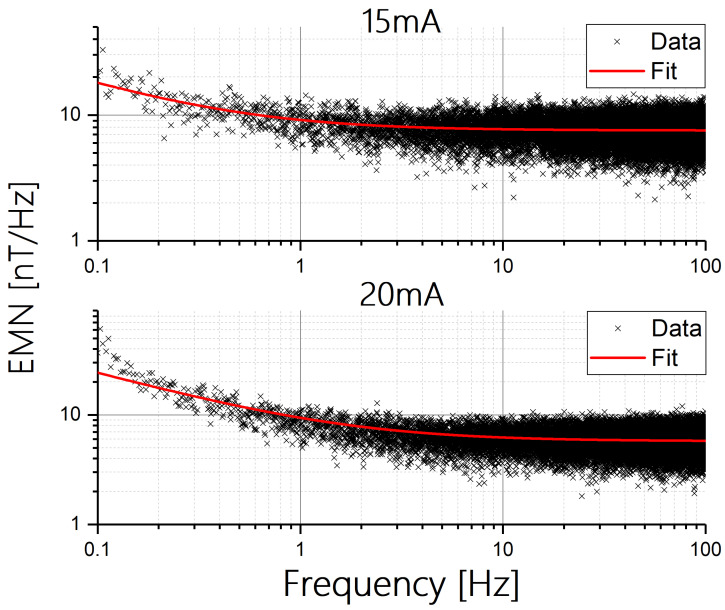
Equivalent magnetic noise (EMN) versus frequency of a PHE sensor with b = 50 microns, measured using currents of 15 mA and 20 mA.

To demonstrate the process of optimizing the current density, we present in Figure 7 the EMN from 0.1 to 100 Hz of a sensor with a hard axis of 50 microns, where the sensor is excited with 15 mA and 20 mA, which are two and three times greater than the current used in Figure 6.

As shown in Figure 7, doubling the current to 15 mA improves the sensor resolution of both low and high frequencies. However, for 20 mA, the resolution does not improve at high frequencies and it becomes worse at low frequencies. We attribute this behavior to detrimental heating effects, as described by us previously [16].

**Figure 8:**
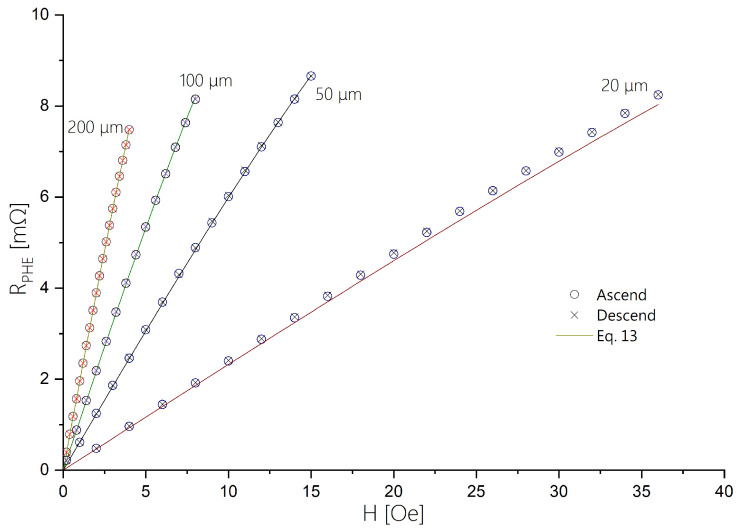
The PHE resistance vs a field applied parallel to the hard axis of the ellipse for sensors with varying hard axis values.

The extended field range of the sensors is manifested in Figure 8, which presents the response of the sensors to a magnetic field applied along the hard axis.

The resistivity of each sensor was measured during both ascending and descending magnetic fields and was also compared, in Figure 8, with the function expected from the Stoner–Wohlfarth model:

(13) $$R_{PHE}=\frac{▵R_{PHE}}{H_{eff}}\cdot H\sqrt{1-{\left(\frac{H}{H_{eff}}\right)}^{2}}$$

where the values of $▵R_{PHE}$ and $H_{eff}$ for each sensor are taken from Table 1.

**Figure 9:**
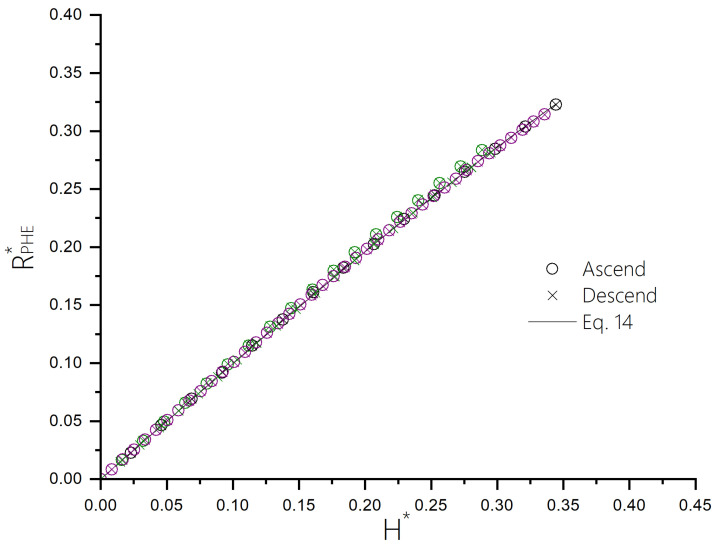
The normalized resistance vs normalized field applied parallel to the hard axis of the ellipse for sensors with varying hard axis values.

Figure 9 shows the scaling of the four data sets presented in Figure 8, with normalized values of $R_{PHE}^{*}=\frac{R_{PHE}}{▵R_{PHE}}$ and $H^{*}=\frac{H}{H_{eff}}$. Hence, $R_{PHE}^{*}$ is given by

(14) $$R_{PHE}^{*}=H^{*}\sqrt{1-{\left(H^{*}\right)}^{2}}$$

We note that the saturation field is $H_{eff}$ and that ideally any field can be applied along the hard axis reversibly. In practice, we find that the single-domain behavior can break and found empirically, for fields lower than $H_{\mathrm{eff}}/3$, that the response is reversible and well described by the model. While the response is intrinsically non-linear, we can calculate the field range where its deviation from linearity is smaller than 1% by using the following formula:

(15) $$\frac{\left|R_{PHE}^{*}-R_{PHE}^{L}\right|}{R_{PHE}^{L}}\leq 0.01$$

where $R_{PHE}^{L}$ is the linear approximation of Equation (14) for a small $H^{*}$ and is given by $R_{PHE}^{L}=H^{*}$. We note that below $H^{*}$∼0.14 the deviation from linearity is less than 1%.

It is evident that although all sensors exhibit non-linear behavior, this behavior can be precisely modeled using the Stoner–Wohlfarth Hamiltonian, which means that they effectively behave as single magnetic domains. Furthermore, Figure 9 illustrates that, upon normalizing the values for each sensor using $\Delta R_{PHE}$ and $H_{\mathrm{eff}}$, all curves align closely. This indicates that the behavior of the sensors is predictable across different hard axis values.

An important feature which affects the performance of magnetic sensors is their hysteresis. Low hysteresis is important for achieving high accuracy and repeatability in measurements. To fully characterize the intrinsic hysteresis of a sensor it is important to perform such measurements with magnetic shielding and with a stabilized temperature. We leave such measurements for future study and present here characterization measurements performed in ambient conditions, where the deviations in room temperature are typically less than ±1 degree.

**Figure 10:**
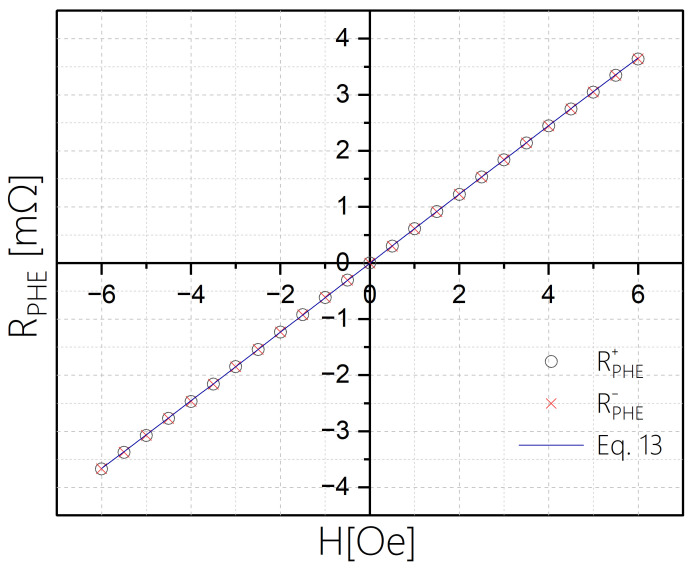
$R_{\mathrm{PHE}}^{+}$ and $R_{\mathrm{PHE}}^{-}$ vs. applied field parallel to the hard axis of the ellipse for a sensor with b=50 microns.

**Figure 11:**
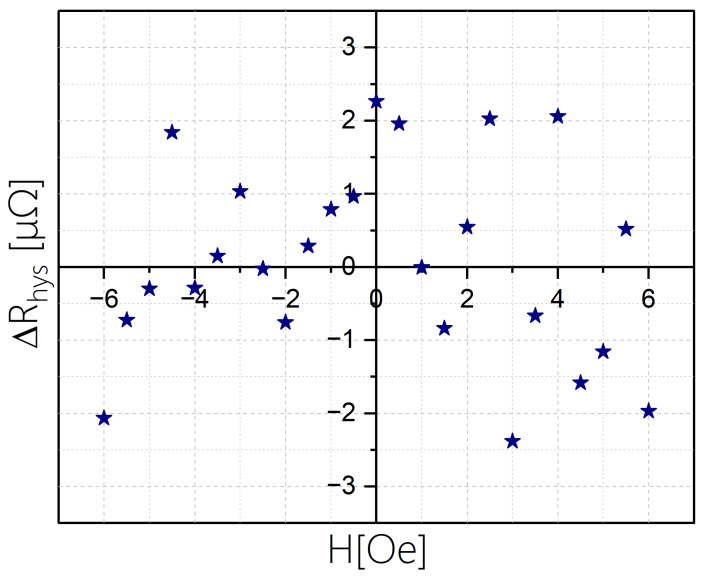
The difference in the resistivity values coming down from 14 Oe or coming up from −14 Oe for a sensor with b=50 microns.

In order to assess the hysteresis of a sensor with b=50 microns, we focus on a field range where its deviation from linearity is less than 1%; namely between −6 Oe and +6 Oe. Using steps of 0.5 Oe we measured $R_{PHE}$ at each field H twice: we first set the field to +14 Oe reduced to H and measured $R_{PHE}$ 10 times (we denoted the average as $R_{PHE}^{+}$). Then, we set the field to −14 Oe increased to H and measured $R_{PHE}$ 10 times (we denoted the average as $R_{PHE}^{-}$). In these measurements, the field is applied parallel to the short axis of the ellipse. Figure 10 shows $R_{PHE}^{+}$ and $R_{PHE}^{-}$ for each field. Additionally, we present in Figure 11 the difference between the two averages for each field, which we denote as $\Delta R_{\mathrm{hys}}=R_{PHE}^{+}-R_{PHE}^{-}$.

The average and standard deviation of $\Delta R_{\mathrm{hys}}$ are 0.67 nΩ and 1.32 μΩ, respectively. Notably, Δ*R*_hys_ changes sign, so clearly the differences are largely unrelated to magnetic hysteresis. Nevertheless, we can consider Δ*R*_hys_ as an upper bound for magnetic hysteresis, if it exists.

Using the linear dependence shown in Figure 8, we can use the value $dR_{PHE}\;/\;dH=6.15\left(\Omega /T\right)$ to convert the $\Delta R_{\mathrm{hys}}$ values shown in Figure 11 using $\Delta B=\frac{\Delta R_{\mathrm{hys}}}{6.15(\Omega /T)}$. Namely, a change of 1 μΩ is achieved by a field change of ~170 nT. We note that while $\Delta R_{\mathrm{hys}}$ fluctuates between positive and negative values on the order of 1 μΩ, the average of $\Delta R_{\mathrm{hys}}$ is two orders of magnitude smaller, so magnetic hysteresis, if it exists, is negligible. If we compare $\Delta R_{\mathrm{hys}}$ to the field range we obtain a hysteresis error of ~$3\cdot 10^{-4}$ and an average hysteresis error of more than an order of magnitude smaller. This is quite encouraging, particularly since it is an upper bound. We associate the negligible hysteresis to the effective single magnetic behavior of the EPHE sensors.

Based on our findings, we can estimate the properties of EPHE with larger magnetic anisotropy fields. Thus, for instance, if an anisotropy field in the kOe range is required, it would be possible to achieve it using sensors with a hard axis of 2 microns. If such a sensor is excited with the same current density (namely, 0.6 mA), we can expect an EMN of about 8 $\mu T/\sqrt{\mathrm{Hz}}$ at frequencies where 1/f noise is largely suppressed. We note that the EMN of the sensors with short axis of 50 and 20 microns was improved by increasing the current density, and this may be the case if the size of the sensors is further decreased. Moreover, as the limiting factor of current density is related to heating, we expect that the performance of all sensors can be improved by adding heat sinks and using active cooling methods.

We note that, in principle, the anisotropy field can be increased by increasing the thickness without detrimental consequences for the EMN. By examining Equation (12), we see that if the current density is maintained, the increase in $I_{x}$ and *t* will cancel out and thus the sensitivity decrease will be proportional to *n* instead of $n^{2}$. However, growing very thick films carries significant challenges, which will be left for future studies.

A prominent example of the use of magnetic sensors is the automotive industry, where they are widely used to enhance vehicle performance, safety, and efficiency. Primarily used for position sensing, they detect the position and speed of critical components like throttle pedals, gear shifters, and brake pedals, enabling the precise operation of engine control systems and transmission mechanisms. Additionally, magnetic sensors are crucial for wheel speed detection, facilitating anti-lock braking systems, and traction control systems, thereby enhancing vehicle stability and safety, especially in challenging road conditions. Moreover, these sensors are employed in current sensing applications, accurately measuring the flow of electrical current in systems such as battery management, power steering, and electric vehicle propulsion systems, contributing to optimal performance and efficiency while ensuring protection against overloads and faults. In addition to the automotive industry, magnetic sensors are widely used in many other industrial applications such as monitoring equipment performance in factories, robotics, factory automation, smart home applications, etc.

The magnetic sensors commonly used in industrial applications are regular Hall effect sensors and magnetoresistive sensors (AMR, GMR, and TMR). Magnetoresistive sensors are typically more sensitive and they are used in two modes. For some applications (e.g., angle sensors) they are used in their saturated state; namely, with fields much larger than the magnetic anisotropy of the sensing element. In others, (e.g., current sensing or positioning) they are used in their linear regime, with field ranges of mT and above. Thus, for example, a recently released magnetoresistive sensor based on TMR technology that is used for proximity and current sensing (CT100 by Allegro) exhibits a field range of 50 mT, an EMN of ~140 $\mathrm{nT}/\sqrt{\mathrm{Hz}}$ at 10 Hz, and a hysteresis error of ~$5\cdot 10^{-4}$. In comparison, our sensor with b=50 microns exhibits an EMN of 7.7 $\mathrm{nT}/\sqrt{\mathrm{Hz}}$ and the upper bound of its hysteresis error is smaller.

We note that, for applications that use magnetoresistive sensors in their saturated state, the EPHE sensors with small anisotropy fields (less than 5 Oe) and $\mathrm{pT}/\sqrt{\mathrm{Hz}}$ EMN can also be considered. Furthermore, this small anisotropy field is advantageous as smaller external magnetic fields can be used for their saturation. For applications where magnetoresistive sensors are currently utilized in their linear regime within a field range of mT and above, we anticipate that this study will facilitate the consideration of EPHE sensors as well. This is because it demonstrates methods to extend the field range of EPHE sensors and presents the anticipated trade-off between field range and EMN.

## 5. Conclusions

In this study, we have demonstrated the feasibility of significantly increasing the operational field range of EPHE sensors and studied the implications of this field range extension on their resolution. Overall, the advancements presented in this study significantly enhance the potential use of EPHE sensors, making them a promising candidate for a wide range of applications requiring precise magnetic field sensing over an extended field range.

The field range increase is achieved by increasing the ratio between the thickness and the short axis of the ellipse, which is the factor that determines the shape-induced uniaxial anisotropy parallel to the long axis of the ellipse. All sensors are of the same thickness and the ratio between their long and short axes was kept constant. The study includes sensors with anisotropy fields ranging from 12 Oe to 120 Oe, with corresponding EMNs at 10 Hz of 800 $\mathrm{pT}/\sqrt{\mathrm{Hz}}$ and 56 $\mathrm{nT}/\sqrt{\mathrm{Hz}}$, respectively.

This study also demonstrates that the studied sensors exhibit effective single magnetic domain behavior and that their hysteresis is negligible, facilitating stable and repeatable sensor performance. These features, together with their extended field range and their relatively good resolution for field ranges exceeding mT, open up new possibilities for the use of EPHE sensors in applications where operational field ranges above mT are essential, such as the automotive industry and industrial automation.

## Figures and Tables

**Table 1:** Typical values of $H_{eff}$, $S_{y}$, $▵R_{PHE}$, $R_{x}$, and $R_{y}$ for EPHE sensors with varying hard axis lengths and uniform current density.

*b* (μm)	I (mA)	$H_{\mathrm{eff}}$ (Oe)	$S_{y}$ (mV/T)	$▵R_{\mathrm{PHE}}$ (mΩ)	$R_{x}$ (Ω)	$R_{y}$ (Ω)
20	3	124.8	7.2	29	8.9	35.6
50	7.5	43.5	46	26	7	20.7
100	15	23.8	151.5	25	5.6	14.4
200	30	13.5	642.1	26	5.1	9.8

## Data Availability

The original contributions presented in the study are included in the article, further inquiries can be directed to the corresponding author.

## References

[1] Fleming W.J. (2001). Overview of automotive sensors. IEEE Sens. J..

[2] Treutler C. (2001). Magnetic sensors for automotive applications. Sens. Actuators A Phys..

[3] Mohankumar P., Ajayan J., Yasodharan R., Devendran P., Sambasivam R. (2019). A review of micromachined sensors for automotive applications. Measurement.

[4] Lim H.S., Kim C.Y. (2022). ISO26262-Compliant Inductive Long-Stroke Linear-Position Sensors as an Alternative to Hall-Based Sensors for Automotive Applications. Sensors.

[5] Heidari H., Nabaei V. (2019). Magnetic Sensors for Biomedical Applications.

[6] Kim S., Torati S.R., Talantsev A., Jeon C., Lee S., Kim C. (2020). Performance validation of a planar hall resistance biosensor through beta-amyloid biomarker. Sensors.

[7] Sinha B., Ramulu T.S., Kim K., Venu R., Lee J., Kim C. (2014). Planar Hall magnetoresistive aptasensor for thrombin detection. Biosens. Bioelectron..

[8] Reininger T., Hanisch C. (1997). Magnetic field sensors for the industrial automation. Sens. Actuators A Phys..

[9] Kim M., Oh S., Jeong W., Talantsev A., Jeon T., Chaturvedi R., Lee S., Kim C. (2020). Highly Bendable Planar Hall Resistance Sensor. IEEE Magn. Lett..

[10] Barroso T.G., Martins R.C., Fernandes E., Cardoso S., Rivas J., Freitas P.P. (2018). Detection of BCG bacteria using a magnetoresistive biosensor: A step towards a fully electronic platform for tuberculosis point-of-care detection. Biosens. Bioelectron..

[11] Wolff J., Heuer T., Gao H., Weinmann M., Voit S., Hartmann U. (2006). Parking monitor system based on magnetic field senso. Proceedings of the 2006 IEEE Intelligent Transportation Systems Conference.

[12] Včelák J., Ripka P., Kubik J., Platil A., Kašpar P. (2005). AMR navigation systems and methods of their calibration. Sens. Actuators A Phys..

[13] Khan M.A., Sun J., Li B., Przybysz A., Kosel J. (2021). Magnetic sensors-A review and recent technologies. Eng. Res. Express.

[14] Mor V., Grosz A., Klein L. (2017). Planar Hall effect (PHE) magnetometers. High Sensitivity Magnetometers.

[15] Nhalil H., Givon T., Das P.T., Hasidim N., Mor V., Schultz M., Amrusi S., Klein L., Grosz A. (2019). Planar Hall effect magnetometer with 5 pT resolution. IEEE Sens. Lett..

[16] Nhalil H., Das P.T., Schultz M., Amrusi S., Grosz A., Klein L. (2020). Thickness dependence of elliptical planar Hall effect magnetometers. Appl. Phys. Lett..

[17] Elzwawy A., Pişkin H., Akdoğan N., Volmer M., Reiss G., Marnitz L., Moskaltsova A., Gurel O., Schmalhorst J.M. (2021). Current trends in planar Hall effect sensors: Evolution, optimization, and applications. J. Phys. D Appl. Phys..

[18] Ejsing L., Hansen M.F., Menon A.K., Ferreira H., Graham D., Freitas P. (2004). Planar Hall effect sensor for magnetic micro-and nanobead detection. Appl. Phys. Lett..

[19] Ejsing L., Hansen M.F., Menon A.K., Ferreira H.A., Graham D.L., Freitas P.P. (2005). Magnetic microbead detection using the planar Hall effect. J. Magn. Magn. Mater..

[20] Granell P.N., Wang G., Cañon Bermudez G.S., Kosub T., Golmar F., Steren L., Fassbender J., Makarov D. (2019). Highly compliant planar Hall effect sensor with sub 200 nT sensitivity. NPJ Flex. Electron..

[21] Pham H.Q., Tran B.V., Doan D.T., Pham Q.N., Kim K., Kim C., Terki F., Tran Q.H. (2018). Highly sensitive planar Hall magnetoresistive sensor for magnetic flux leakage pipeline inspection. IEEE Trans. Magn..

[22] Nhalil H., Schultz M., Amrusi S., Grosz A., Klein L. (2022). High sensitivity planar hall effect magnetic field gradiometer for measurements in millimeter scale environments. Micromachines.

[23] Lim J., Sinha B., Ramulu T.S., Kim K., Kim D.Y., Kim C. (2013). NiCo sensing layer for enhanced signals in planar hall effect sensors. Met. Mater. Int..

[24] Elzwawy A., Kim S., Talantsev A., Kim C. (2019). Equisensitive adjustment of planar Hall effect sensor’s operating field range by material and thickness variation of active layers. J. Phys. D Appl. Phys..

[25] Cao Z., Chen W., Lu S., Yan S., Zhang Y., Zhou Z., Yang Y., Li Z., Zhao W., Leng Q. (2021). Tuning the linear field range of tunnel magnetoresistive sensor with MgO capping in perpendicular pinned double-interface CoFeB/MgO structure. Appl. Phys. Lett..

[26] Mor V., Schultz M., Sinwani O., Grosz A., Paperno E., Klein L. (2012). Planar Hall effect sensors with shape-induced effective single domain behavior. J. Appl. Phys..

[27] Goto M., Tange H., Kamimori T. (1986). Thickness dependence of field induced uniaxial anisotropy in 80-permalloy films. J. Magn. Magn. Mater..

[28] Grosz A., Mor V., Paperno E., Amrusi S., Faivinov I., Schultz M., Klein L. (2013). Planar Hall Effect Sensors with Subnanotesla Resolution. IEEE Magn. Lett..

[29] Grosz A., Mor V., Amrusi S., Faivinov I., Paperno E., Klein L. (2016). A High-Resolution Planar Hall Effect Magnetometer for Ultra-Low Frequencies. IEEE Sens. J..

[30] Gehanno V., Freitas P.P., Veloso A., Ferrira J., Almeida B., Soasa J., Kling A., Soares J., Da Silva M. (1999). Ion beam deposition of Mn-Ir spin valves. IEEE Trans. Magn..

[31] Park E.B., Jang S.U., Kim J.H., Kwon S.J. (2012). Induced magnetic anisotropy and strain in permalloy films deposited under magnetic field. Thin Solid Film..

